# Analysis of TCR Repertoire by High-Throughput Sequencing Indicates the Feature of T Cell Immune Response after SARS-CoV-2 Infection

**DOI:** 10.3390/cells11010068

**Published:** 2021-12-27

**Authors:** Yifan Wang, Fugang Duan, Zhu Zhu, Meng Yu, Xiaodong Jia, Hui Dai, Pingzhang Wang, Xiaoyan Qiu, Yinying Lu, Jing Huang

**Affiliations:** 1 Department of Immunology, School of Basic Medical Sciences, Peking University, 38 Xueyuan Road, Haidian District, Beijing 100191, China; wangyifanpku@pku.edu.cn (Y.W.); dfg5535@126.com (F.D.); jujuonfire@yeah.net (Z.Z.); 1711110028@pku.edu.cn (M.Y.); daihui@bjmu.edu.cn (H.D.); wangpzh@bjmu.edu.cn (P.W.); 2 NHC Key Laboratory of Medical Immunology, Peking University, Beijing 100191, China; 3 Key Laboratory of Molecular Immunology, Chinese Academy of Medical Sciences, Beijing 100191, China; 4 Comprehensive Liver Cancer Center, The 5th Medicine Center of PLA General Hospital, Beijing 100039, China; feixiang.5420@163.com

**Keywords:** SARS-CoV-2, COVID-19, T cell receptor, CDR3, immune memory

## Abstract

Coronavirus disease 2019 (COVID-19) is a global infectious disease caused by the SARS-CoV-2 coronavirus. T cells play an essential role in the body’s fighting against the virus invasion, and the T cell receptor (TCR) is crucial in T cell-mediated virus recognition and clearance. However, little has been known about the features of T cell response in convalescent COVID-19 patients. In this study, using 5′RACE technology and PacBio sequencing, we analyzed the TCR repertoire of COVID-19 patients after recovery for 2 weeks and 6 months compared with the healthy donors. The TCR clustering and CDR3 annotation were exploited to discover groups of patient-specific TCR clonotypes with potential SARS-CoV-2 antigen specificities. We first identified CD4^+^ and CD8^+^ T cell clones with certain clonal expansion after infection, and then observed the preferential recombination usage of V(D) J gene segments in CD4^+^ and CD8^+^ T cells of COVID-19 patients with different convalescent stages. More important, the TRBV6-5-TRBD2-TRBJ2-7 combination with high frequency was shared between CD4^+^ T and CD8^+^ T cells of different COVID-19 patients. Finally, we found the dominant characteristic motifs of the CDR3 sequence between recovered COVID-19 and healthy control. Our study provides novel insights on TCR in COVID-19 with different convalescent phases, contributing to our understanding of the immune response induced by SARS-CoV-2.

## 1. Introduction

Beginning in December 2019, the coronavirus disease 2019 (COVID-19) outbreak has posed a serious threat to more than 200 countries worldwide and has caused more than 5.2 million deaths (https://covid19.who.int. accessed on 13 December 2021). COVID-19 is caused by severe acute respiratory syndrome coronavirus-2 (SARS-CoV-2), which usually leads to respiratory infections, and severe cases develop into severe pneumonia or even death [1,2,3]. The immune response to COVID-19 encompasses both B cell-mediated humoral responses through antibodies as well T cell activity. Many of the early studies on the immune response to SARS-CoV-2 have focused on neutralizing antibodies. A growing body of evidence suggests T cell responses are important for both early viral clearance as well as conferring protection through memory T cells for extended periods in COVID-19 patients [4,5,6,7,8]. However, the specific T cell immune response against SARS-CoV-2, including the underlying mechanisms, remains unclear.

The cellular immune response is mediated by T cells, which play a role in the direct killing of virus-infected cells via CD8^+^ cytotoxic T cells as well as helping to direct the overall immune response through CD4^+^ helper T cells. The humoral immune response also includes CD4^+^ T cells that assist B cells in differentiating into plasma cells and subsequently producing antibodies specific to the viral antigen. CD4^+^ T cells also promote the immune response of CD8^+^ T cells and the formation of long-term memory CD8^+^ T cells to exert antiviral capabilities effectively [9]. Thus, T cells are an important target for assessing the degree of SARS-CoV-2 infection and disease progression [10]. The T cell response to SARS-CoV-2 peaks about one to two weeks after infection and can be detected during the months of recovery [11]. It has been reported that the activated CD38^+^HLA-DR^+^, CD8^+^ T cells, and CD38^+^HLA-DR^+^ CD4^+^ T cells that respond to virus infection are transiently increased in COVID-19 patients [12]. Significant elevation and unusual phenotypes of CD4^+^ cells, which exhibit both a proliferative exhausted phenotype and a clonally expanded cytotoxic phenotype, were also observed in mild and moderate COVID-19 cases. However, these phenotypes between these two groups of CD4^+^ T cells of mild and moderate COVID-19 cases exhibit distinct functional signatures, distinct TCR sharing patterns, and may represent two divergent destinations for naive CD4^+^ T cells [13]. Moreover, a higher proportion of SARS-CoV-2 specific CD8^+^ T cells were detected in mild cases, and these CD8^+^ T cells have extensive and strong memory after the recovery period of COVID-19 [14]. On the contrary, a significant reduction in effector memory CD4^+^ T cells, which were less expanded and skewed toward central memory T cells and TH2-like phenotypes, was detected in COVID-19 patients with severe disease, whereas terminally differentiated CD8^+^GZMK^+^ effector cells were clonally expanded both during and after the infection [15]. The CD4^+^ T memory response was detected in all recovered patients from COVID-19, and 70% of patients had established CD8^+^ T memory response to SARS-CoV-2, lasting for more than 2 months [16,17]. Additionally, in COVID-19-recovered individuals receiving the vaccine, pre-existing SARS-CoV-2 specific memory cells showed both clonal expansion and a phenotypic shift towards more differentiated CCR7^−^CD45RA^+^ effector cells. Therefore, memory T cells have always been the focus of attention after SARS-CoV-2 infection.

T cells recognize pathogen-derived peptides presented with the major histocompatibility complex (MHC) on the cell surface using hypervariable T cell receptor (TCR). TCR diversity is widely recognized as a direct measure of immune competence, as it quantifies the variety of foreign antigens and hence acts upon them. At the early stage of T cell development, TCR is generated by somatic rearrangement of variable (V), diversity (D), and joining (J) gene segments, known as V(D)J recombination [18]. Thus, the TCR repertoire is a critical factor in viral clearance. After activation by antigen recognition, T cells undergo clonal expansion, during which activated T cells rapidly proliferate to generate large numbers with identical TCRs to eliminate virus-infected cells. Most of the TCRs on circulating T cells are alpha and beta subunit heterodimers, and the specificity for an antigen is shaped by VDJ recombination [19].

Recently, sequencing technology has been widely used to reveal activation-induced phenotypic profiles of antigen-reactive T cells [20,21]. Some studies have revealed that clonality and skewing of TCR repertoires from COVID-19 patients by next-generation sequencing were associated with severity of diseases, such as early CD4^+^ and CD8^+^ T cell activation and interferon type I and III responses. The emergence of shared T cell clusters occurs in the rehabilitation of COVID-19 patients by next-generation sequencing technology [22]. It has also been reported that the patients with moderate COVID-19 have highly clonally expanded CD8^+^ T cells [23]. Notably, the memory phenotype of T cell response is positively correlated with the severity of the disease, and there exists the specific memory CD8^+^ T cell in convalescent COVID-19 patients by single-cell sequencing [24]. Additionally, preferential usage of V and J gene segments of TCR was also observed in COVID-19 patients [25]. However, studies on the TCR repertoire of COVID-19 patients with different convalescent stages are lacking. Further exploration is essential to understand the mechanism of T cell response to SARS-CoV-2 and the formation of SARS-CoV-2-specific memory T cells.

In this study, we used the 5′RACE (Rapid Amplification of cDNA Ends) method combined with third-generation high-throughput sequencing technology to investigate the TCRα and TCRβ library sequences on peripheral blood CD4^+^ and CD8^+^ T cells of convalescent patients after SARS-CoV-2 infection, and then further analyzed the characteristics of their TCR repertoire of convalescent patients with different convalescent stages.

## 2. Materials and Methods

### 2.1. Donors and Blood Samples

Nine confirmed COVID-19 patients of 2 weeks convalescence and five healthy controls were obtained from Fifth Medical Center of Chinese PLA General Hospital, Beijing, China. The clinicopathologic characteristics of the study group participants with 2 weeks convalescence and healthy controls are listed in Table 1. Additionally, twenty confirmed COVID-19 patients of 6 months convalescence were obtained from the General Hospital of Central Theater Command of PLA, Wuhan, China. The study protocol was approved by the Institutional Review Board of Peking University (PUIRB). We complied with all relevant ethical regulations, and informed consent was obtained from all human participants (No:2020072D). No statistically significant difference in age or sex was observed among the three groups of patients.

### 2.2. Isolation of PBMCs and T Cell Subpopulations

PBMC were isolated with the Ficoll-Paque density gradient centrifugation protocol. The CD4^+^ T and CD8^+^ T cells were purified from PBMCs with the Easysep^®^ human CD4^+^ or CD8^+^ positive selection kits from Stem Cell technologies (San Diego, CA, USA). Purified cells were eluted and washed with PBS containing 2% (*v*/*v*) fetal bovine serum (FBS) and 1mM/L EDTA.

### 2.3. TCRα and TCRβ Library Preparation and PCR Amplification

For the sorted CD4^+^ T and CD8^+^ T cells, total RNA was extracted using RaPure Total RNA Micro Kit (Magen, China) according to the manufacturer’s instructions. A universal primer binding site, sample barcode, and unique molecular identifier (UMI) sequences were introduced using the SMARTer^®^ RACE 5′/3′ kit (Takara, Japan) with TCRα and TCRβ constant segment-specific primers (Forward: CTAATACGACTCACTATAGGGC; Reverse1: GATTACGCCAAGCTTCACGGCAGGGTCAGGGTTCTGGATAT; Reverse 2: GATTACGCCAAGCTTCTCGGGTGGGAACACGTTTTTCAGGTCCTC) for cDNA synthesis. cDNA libraries were amplified in two PCR steps, introducing the second sample barcode and Illumina TruSeq adapter sequences at the second PCR step. The PCR program for both rounds was: 5 cycles at 94 °C for 30 s, 5 cycles at 68 °C for 30 s, and 25 cycles at 72 °C for 3 min (first-round PCR) and 40 cycles at 94 °C for 30 s, 68 °C for 30 s, and 72 °C for 2 min (second-round PCR). The PCR products were separated on 1.5% agarose gel by electrophoresis.

### 2.4. TCRα and TCRβ Sequencing and Data Preprocessing

We measured the concentration of PCR products with a barcode and mixed the same amount of PCR products from all COVID-19 patients and healthy controls. The mixed PCR products were separated with 2% agarose gel, and then the extracted DNA was sent to Novogene Institute (Beijing, China). The amplicons were sequenced on a PacBio Sequel system using V3 chemistry (Courtesy of Pacific Biosciences of California Inc., Menlo Park, CA, USA). The raw data (raw reads) in FASTQ format were first processed using Python and custom Perl scripts. Clean data (clean reads) were obtained after removing various reads, including poly-N reads, reads without a 3′ adapter or an insert tag, reads with 5′ adapter contamination, reads with poly-A, T, G, or C low-quality reads from the raw data. Moreover, the Q20, Q30, and GC contents of the raw data were calculated. All sequences shorter than 200 bps, having homopolymers of 6 bps barcodes, containing primer mismatches, and with a quality score lower than 19, were removed.

### 2.5. TCR Repertoire Analysis

The processing stage of TCR repertoire analysis started with the mapping of V, D, and J segments. Filtered reads were subsequently aligned to V, D, J, and C gene segments of TCR alpha (TRA) and TCR beta (TRB) locus for clonotype assembly of complementarity-determining region 3 (CDR3) nucleic acid sequences by IMGT/HighV-QUEST web server. Furthermore, the rarefaction curve of the TCR repertoire was generated using VDJtools for each subject. We obtained the core tabular format for VDJ tools by the conversion supported in the program, which contained count, frequency, CDR3 nucleotide sequence, Vend, D start, D end, and J start. The frequency of various V–J junctions was calculated and displayed using basic analysis of VDJtools, making circus-style V–J usage plots. Additionally, the diversity estimation was utilized to compute a set of diversity statistics as well as to visualize the repertoire clonality and the comparison of diversity estimates. The V(D)J patterns of each sample were combined to analyze the dominant rearrangement pattern in different groups. The CDR3nt and CDR3aa in advantageous V(D)J usage were merged to further search for the dominant CDR3. All statistical analysis was implemented with R software (Version 4.1.0, downloaded from http://www.r-project.org, accessed on 10 November 2021).

## 3. Results

### 3.1. Clinical Characteristics of COVID-19 Patients with Different Convalescent Stages for TCR Repertoire Profiling Analysis

To evaluate the blood immune characteristics of COVID-19 patients with different convalescent stages, thirty-four samples were included in the current study, including nine COVID-19 patients with two-week convalescent stage, twenty COVID-19 patients at the six-month-convalescent stage, and five healthy controls (Table 1). Moreover, these COVID-19 patients were classified into three groups, including moderate, severe, and critical cases. As shown in Table 1, the analysis of the blood routine test showed the number of white blood cells, mainly neutrophil, increased in the severe and critical cases of COVID-19 patients at 2 weeks convalescence compared with healthy controls. However, the percentage and absolute number of lymphocytes and monocytes obviously reduced in the severe and critical cases compared with healthy controls. In addition, C-reactive protein (CRP) and creatine kinase (CK) were also elevated in the severe and critical cases, which had complications, including ARDS but not secondary infection.

**Figure 1:**
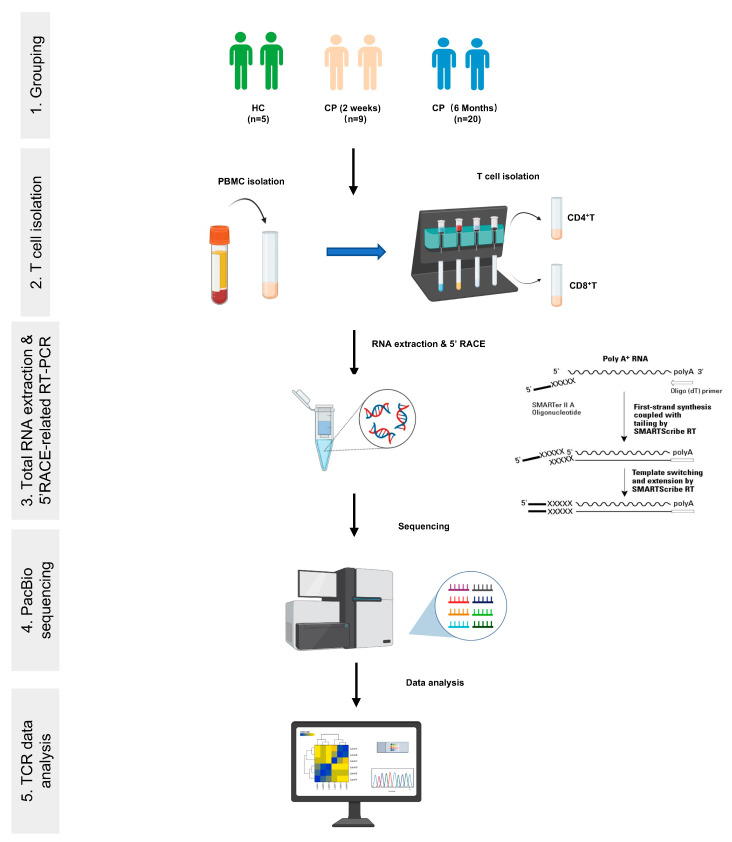
Schematic diagram of TCR repertoire analysis in the convalescent COVID-19 patients. Peripheral blood mononuclear cells were collected from COVID-19 patients with 2-week and 6-month convalescence phase and healthy controls, and CD4^+^ T and CD8^+^ T cells were sorted to perform 5′ RACE-related RT-PCR combined with PacBio sequencing.

We next collected their peripheral blood samples and isolated peripheral blood mononuclear cells (PBMCs). For each blood sample, we purified CD4^+^ T cells and CD8^+^ T cells with magnetic beads, respectively. From all samples, we isolated RNA and performed 5′RACE coupled with long-read high-throughput sequencing to amplify the complete DNA sequence of TCRα (TRA) and TCRβ (TRB) chains of antigen-specific T cells (Figure 1). A total of 32,796 and 15,086 TCRα and β chain sequences were obtained from COVID-19 patients and healthy controls, respectively. Among them, there are 11,324 effective TRA sequences and 6933 TRB sequences detected in CD4^+^ T cells. There are 21,472 effective TRA sequences and 8153 TRB sequences detected in CD8^+^ T cells (Table 2).

### 3.2. Significance of T Cell Receptor Bias during the Different Convalescent Phases of COVID-19 Patients Compared with Healthy Controls

**Figure 2:**
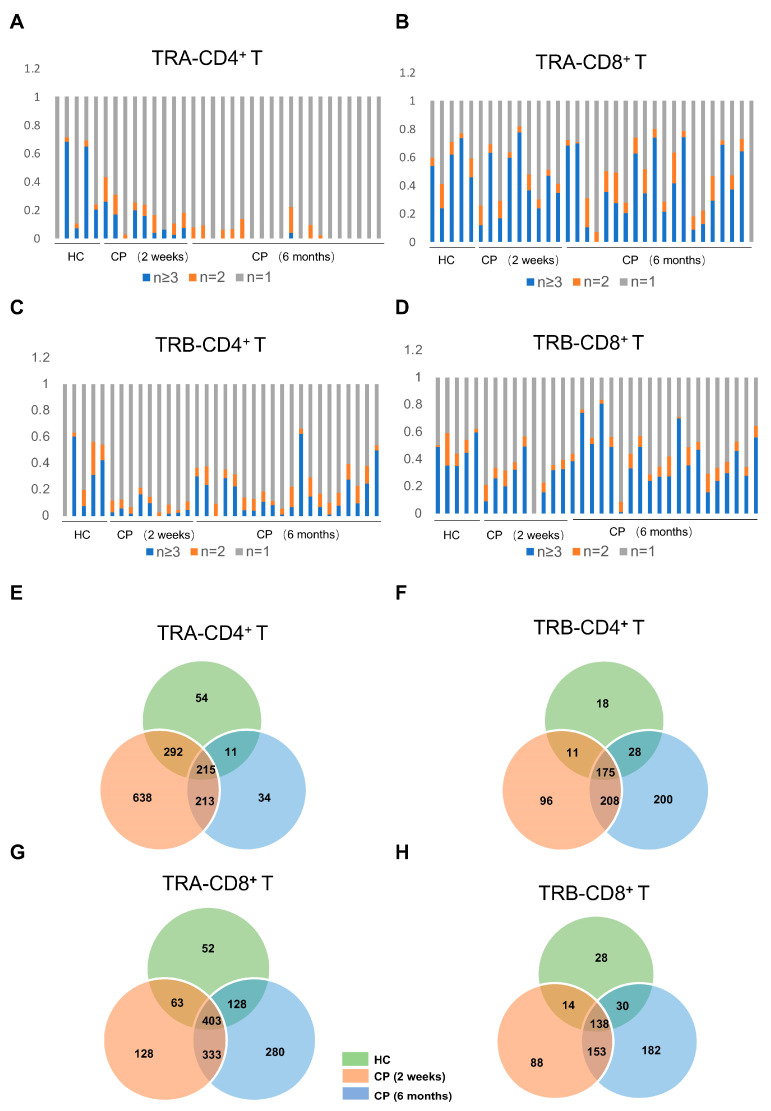
The types of TCRα (TRA) and TCRβ (TRB) clones from CD4^+^ T and CD8^+^ T cells of COVID-19 patients with 2-week and 6-month convalescence phase and healthy controls are shown (**A**–**D**). The TCR distribution TRA (**A**,**B**) and TRB (**C**,**D**) in CD4^+^ T and CD8^+^ T cells of convalescence COVID-19 patients (CP) and healthy controls (HC) are shown. Unique (n = 1), duplicated (n = 2), and clonal (n ≥ 3) TCRs are marked with different colors (**E**–**H**). The Venn diagram shows that the common and specific TRA (**E**,**G**) and TRB (**F**,**H**) numbers in CD4^+^ T and CD8^+^ T cells of convalescence COVID-19 patients (CP) and healthy controls (HC).

To investigate the changes in TCR repertoire after COVID-19 infection, we performed a comparative analysis of the T cell repertoire between the COVID-19 patients at different convalescent phases and healthy controls. Profiling of TRA and TRB repertoire showed that there is more diverse TCR clonality in CD8^+^ T cells compared with CD4^+^ T cells. At the same time, no significant difference was observed in overall frequencies of abundant TRA and TRB clonotypes in convalescent COVID-19 patients, relative to healthy controls (Figure 2A–D), possibly because the clonality frequency of TCR was not caused directly by SARS-CoV-2 infection. Moreover, certain TCRs were shared between the convalescent COVID-19 patients and healthy controls, while more TCRs were shared between the COVID-19 patients at the two-week convalescent stage and six-month convalescent stage (Figure 2E–H).

**Figure 3:**
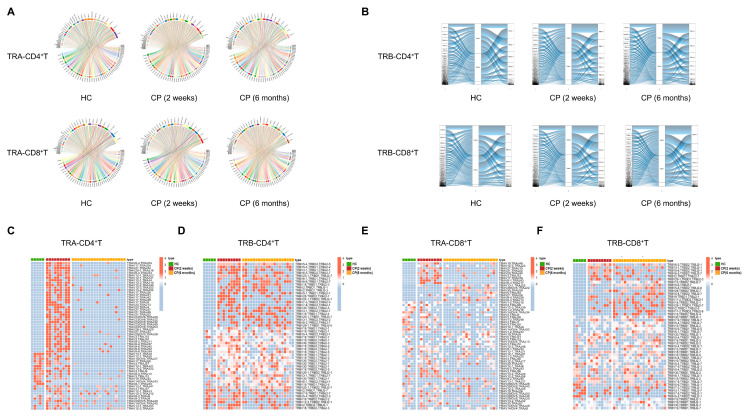
The V, D, and J gene usage comparison of TCRα (TRA) and TCRβ (TRB) chain in the convalescent COVID-19 patients. (**A**) The Circos plots show the difference between CD4^+^ T cell TRA-VJ (top) and CD8^+^ T cell TRA-VJ (bottom) among different COVID-19 patients with 2-week and 6-month convalescence phase (CP) and healthy controls (HC). (**B**) Sankey diagram shows the recombinant pattern of TRB VDJ in the CD4^+^ T (top) and CD8^+^ T cell (bottom) among different convalescence COVID-19 patients (CP) and healthy controls (HC). (**C**–**F**) A histogram of V(D)J gene recombinant pattern of TRA and TRB in CD4^+^ T and CD8^+^ T cells of COVID-19 patients (CP) with 2-week and 6-month-convalescence phase compared with healthy controls (HC). (**C**) TRA in CD4^+^ T cells. (**D**) TRB in CD4^+^ T cell. (**E**). TRA in CD8^+^ T cells. (**F**) TRB in CD8^+^ T cell.

T cell receptors are generated by rearrangement of V and J gene segments for the TRA and by V, D, and J gene segments for the TRB [22]. Here, we explored the usage bias of V, D, and J gene segments for convalescent COVID-19 patients. First, we found that some V, D, and J gene segments on the TRA and TRB were significantly more frequent than healthy controls. Among convalescent COVID-19 patients, for TRA, the most frequently used gene segments were TRAV12-3 and TRAJ42 in either CD4^+^ T or CD8^+^ T cells (Figure 3A), while the frequencies of TRBV23-1 and TRBJ2-7 were significantly higher for TRB (Figure 3B).

To further explore whether a unique V(D)J recombination pattern is specific for convalescent COVID patients, we next compared the V(D)J paring of TRA and TRB in each individual separately. In the CD4^+^ T cells of COVID-19 patients at the two-week convalescent stage, 37 unique pairs of VJ rearrangement of TRA were found, such as TRAV8-4/TRAJ54 and TRAV17/TRAJ54 (Figure 3C), and 23 specific pairs of TRB VDJ patterns were also found in CD4^+^ T cells (Figure 3D). Similar results were obtained in CD8^+^ T cells, which showed there were 16 individual pairs of TRA VJ patterns and 18 specific pairs of TRB VDJ patterns in CD8^+^ T cells of convalescent COVID-19 patients (Figure 3E,F). Of note, these unique gene pairs of TRB, such as TRBV5-4/D2/TRBJ2-5 and TRBV5-4/D1/TRBJ2-2, appeared at the 2-week convalescent stage and continued to the 6-month convalescent stage (Figure 3D,F), suggesting the clones with these TRB VDJ patterns might represent memory T cells phenotype. They may have expanded and participated in the elimination of residual SARS-CoV-2 in the convalescence stage. More important, we found some unique VDJ recombination of TRB was shared in both CD4^+^ T cells and CD8^+^ T cells of two-week convalescent COVID-19 patients, including TRBV6-5/D2/TRBJ2-7 and TRBV2/D1/TRBJ2-1, suggesting that these T cell clones with the above VDJ recombination were specific for SARS-CoV-2 antigen (Figure 3D,F). Additionally, these specific pairs of TRA and TRB VDJ patterns in CD4^+^ T or CD8^+^ T cells existed among the three groups, including moderate, severe, and critical cases, suggesting that these sequences were not significantly correlated with disease severity (data not shown).

### 3.3. CDR3 Sequence Motifs of Responding Clones

**Figure 4:**
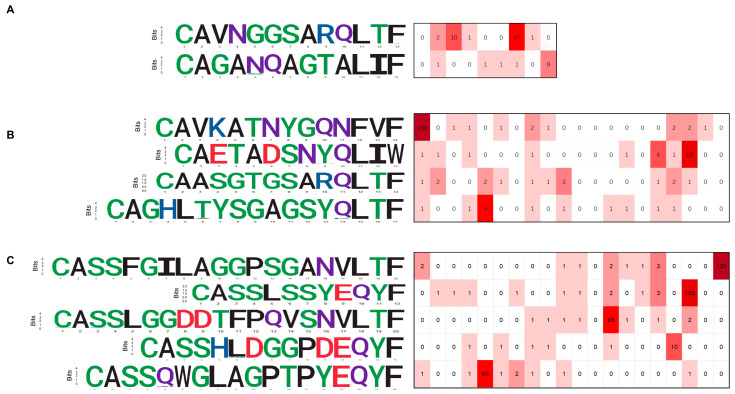
The unique TCR sequence motif and epitope specificity of CD8^+^ T cell clones in COVID-19 patients with 2-week and 6-month convalescence phase. (**A**) The dominant CDR3 motif of TRA in COVID-19 patients with 2-week convalescence phase. (**B**) The dominant CDR3 motif of TRA in COVID-19 patients with 6-month convalescence phase. (**C**) The dominant CDR3 motif of TRB in COVID-19 patients with 6-month convalescence phase.

It was previously shown that TCRs that recognize the same antigen usually have highly similar TCR sequences [26], and the complementarity determining region 3 (CDR3) motif in the TCR component sequence plays an important role [27]. Moreover, the above data revealed potential SARS-CoV-2-specific and highly shared TCR recombination in CD4^+^ T cells and CD8^+^ T cells of convalescent COVID-19 patients. Therefore, we identified the dominant CDR3 sequences in each group. Due to the highly diverse nature of CDR3, only a few consensus sequences were identified, of which two CDR3 sequences of TRA were shared in CD8^+^ T cells of two-week convalescent COVID-19 patients (Figure 4A), as well as four CDR3 sequences of TRA and five CDR3 sequences of TRB that were shared in CD8^+^ T cells of six-month convalescent COVID-19 patients (Figure 4B,C). In contrast, no identical CDR3 sequences were found in all CD4^+^ T cells of different convalescent COVID-19 patients. Compared with the published SARS-CoV-2-related TCR clusters, similar CDR3 motifs have not been reported, suggesting that these might be potential SARS-CoV-2-specific CDR3 motifs. Taken together, our results indicate that the convalescent COVID-19 patients had undergone distinct T cell responses during SARS-CoV-2 viral infection.

## 4. Discussion

To date, the T cell immune regulation mechanism of COVID-19 is still unclear. In this study, we used 5′ RACE combined with the third-generation high-throughput sequencing technology to confirm (a) the changes in TCR clones during the different convalescent phases of COVID-19 and the priority use of V and J gene fragments in COVID-19 patients compared with healthy controls; (b) there is persistent TCR dominant V(D)J recombination during the convalescent phase of COVID-19 from 2 weeks to 6 months; (c) the unique CDR3 motif specific for SARS-CoV-2 in CD8^+^ T cells at the convalescent phase in COVID-19 patients. This finding is in line with other recent studies, where preferential usage of V and J gene segments in convalescent COVID-19 patients was found to be specific to SARS-CoV-2 antigens, suggesting a T cell-mediated immune response to achieve virus clearance. More important, we identified the dynamics of both CD4^+^ T and CD8^+^ T cell responses at different convalescent phases, showing the potential immune memory phenotype, which helps us understand the immune response induced by SARS-CoV-2 infection.

In recent years, TCR repertoire in various diseases was characterized using TCR-seq. Multiplex PCR-, target enrichment-, and 5′ RACE (RNA only)-based approaches are the most widely used TCR-seq strategies. When using multiplex PCR, preferential amplification of highly abundant gene products can occur, contributing to inaccuracies in reported clone frequencies. Additionally, intronic sequences and incomplete VDJ recombination products may contribute to data noise when using gDNA samples. Target enrichment strategies reduce the extent of amplification bias seen in standard multiplex PCR but are more labor/time-intensive and do not resolve the issues associated with gDNA input [28]. However, the 5′ RACE approach avoids the PCR bias seen in the above two methods. It achieves equally efficient amplification of all TCR transcripts regardless of abundance when combined with sequencing platforms capable of long reads, such as the illumine Miseq, as fragment size can exceed 600 bp [29]. Currently, single-cell TCR-seq in tandem with single-cell flow cytometry sorting is an approach for precisely identifying SARS-CoV-2-specific T cells, while it can be limited by their depth and their ability to capture minor clones. Therefore, in our study, we applied 5′ RACE technology combined with PacBio Sequel system capable of long reads (10,000 bp) as third-generation sequencing technology to obtain a wealth of TCR sequences, which effectively avoided the sequence tendency caused by traditional multiple PCR and retained the accuracy of sequencing to the greatest extent. Therefore, the method we adopted more truly and comprehensively demonstrated the characteristics of TCR.

The TCR is composed of TCRα and TCRβ chains, which containing VJ and VDJ gene segments, separately [30]. The results of single-cell sequencing by Wang et al. have reported that the highest frequency of TCR sequence recombination during the convalescent phase of COVID-19 is TRAV12-2-J27-TRBV7-9-J2-3 [25]. Alina et al. had found that TRAV12-1 and TRBV7-9 were used by 71% and 16% of SARS-CoV-2 YLQ-epitope-specific TCRs, and TRAV13-2 and TRBV6-5 were used by 15% and 25% of SARS-CoV-2 RLQ-epitope-specific TCRs, compared with just 3–4% gene usage in control TCRs [4]. Similarly, our results also show the preferential V(D)J sequences only existing in the convalescent COVID-19 patients, including TRAV8-4/TRAJ54 and TRAV17/TRAJ54, TRBV5-4/D2/TRBJ2-5, and TRBV5-4/D1/TRBJ2-2, which were different from public SARS-CoV-2-specific TCRs. Combinations of TCRα and TCRβ genes were highly sample-specific between different COVID-19 patients and healthy controls. A larger population cohort is urgently needed to provide more statistical T cell receptor preference. Interestingly, our results also revealed that identical VDJ sequences, such as TRBV6-5/D2/TRBJ2-7 and TRBV2/D1/TRBJ2-1, were shared by CD4^+^ T cells and CD8^+^ T cells. Although CD4^+^ T and CD8^+^ T cells exhibit their roles differently, CD8^+^ T cells recognize and kill virus-infected cells through TCR-mediated viral antigens [31], while CD4^+^ T cells have multiple roles in coordinating and mediating immune responses against viruses [32]. Therefore, our data suggest that cross-reactive CD4^+^ T and CD8^+^ T cells can participate in the joint resistance to SARS-CoV-2 infection via the production of a virus-specific TCR recombination pattern.

In the process of V(D)J recombination, random nucleotide deletions and insertions often occur in the CDR3, which is essential for antigen binding [33]. Therefore, this process produces a large number of recombinant TCRs, and the differences in CDR3 sequences are usually used to characterize essential indicators of the immune repertoire. We also identified a few consensus CDR3 sequences shared in CD8^+^ T but not CD4^+^ T cells of convalescent COVID-19 patients. Moreover, no identical CDR3 motifs have not been reported relative to the published SARS-CoV-2-related TCR clusters. Our results suggest that these CD8^+^ T cells with unique SARS-CoV-2-associated CDR3 motifs might play a crucial role in the immune response to eliminate the SARS-CoV-2 virus efficiently. However, these unique T cell sequence motifs require prospective validation to be used in COVID-19 patients, and specific T cells reactive to SARS-CoV-2 epitopes also need to be identified in the future. Therefore, our TCR data might help to fingerprint a shared clonal expression T cell phenotype that existed within a population of COVID-19 patients and that defined anti- SARS-CoV-2 immunity in individuals.

Furthermore, T cells with long-lasting memory have been detected in patients recovered from SARS-CoV-2 infection [7], and SARS-CoV-2-specific T cells exhibit a multifunctional memory phenotype during the convalescent phase [34]. In our study, we also found that some particular TRB VDJ recombination patterns in CD4^+^ T and CD8^+^ T cells, such as TRBV5-4/D2/TRBJ2-5 and TRBV5-4/D1/TRBJ2-2, appear at the 2-week convalescent stage and continue to the 6-month convalescent stage, supporting that these T cell clones might have long-term memory characteristics after SARS-CoV-2 infection to participate in the elimination of residual SARS-CoV-2 in the convalescence stage.

## 5. Conclusions

Our findings reveal that COVID-19 patients have unique cellular immune characteristics during the different convalescent phases, which contributes to our understanding of the immune response induced by SARS-CoV-2 and might be used as a basis for prognostic evaluation and targeted therapy for COVID-19 patients.

## Figures and Tables

**Table 1:** Clinicopathologic characteristics of the study population.

	HC (*n* = 5)	CP (2 Weeks) (*n* = 9)		
**Severity**	Healthy (*n* = 5)	Moderate (*n* = 3)	Severe (*n* = 3)	Critical (*n* = 3)
**Age (years)**	65.4 ± 8.76	58 ± 12.78	67 ± 9.9	73 ± 5.57
**Sex**				
Male	2	1	1	2
Female	3	2	2	1
**White blood cells (10^9^/L)**	5.83 ± 1.2	4.97 ± 1.35	7.76 ± 4.87	7.2 ± 3.68
**Percentage of neutrophil**	60.48 ± 6	59.3 ± 11.47	79.3 ± 2.4	90 ± 6.9
**Number of neutrophils (10^9^/L)**	3.22 ± 0.6	3.1 ± 1.27	6.11 ± 3.68	7.29 ± 3.7
**Percentage of lymphocytes**	20.5 ± 10.2	32.7 ± 11.46	16.4 ± 0.7	5.97 ± 4.5
**Number of lymphocytes (10^9^/L)**	1.6 ± 0.92	1.49 ± 0.23	1.26 ± 0.74	0.36 ± 0.04
**Percentage of monocytes**	6.27 ± 0.84	6.48 ± 0.45	4 ± 3.39	2.85 ± 2.69
**Number of monocytes (10^9^/L)**	0.28 ± 0.03	0.32 ± 0.08	0.4 ± 0.46	0.24 ± 0.03
**CRP (mg/L)**	1.91 ± 2.4	2.89 ± 1.1	8.49 ± 10.8	44.89 ± 39
**CK (U/L)**	25 ± 5.4	39.8 ± 16.46	35.5 ± 14.8	133.7 ± 100
**Alkalosis**	N	N	Y (2)	Y (3)
**Complication**				
ARDS	N	N	Y (2)	Y (3)
Secondary infections	N	N	N	N
**History of smoking**	N	N	N	N

HC, healthy controls; CP (2 weeks), COVID-19 patients with 2-week convalescence phase; CP (6 months), COVID-19 patients with 6-month convalescence phase; CRP, C-reactive protein; CK, creatinine kinase; ARDS, acute respiratory distress syndrome. N, none; Y, yes. The parentheses indicate the number of patients.

**Table 2:** The total number of TCR α and β chain sequences were obtained.

	CD4^+^ T			CD8^+^ T			Total
	HC	CP (2 Weeks)	CP (6 Months)	HC	CP (2 Weeks)	CP (6 Months)	
**TRA**	3372	7298	654	4125	6670	10,677	32,796
**TRB**	951	2066	3916	805	1820	5528	15,086

HC, healthy controls; CP (2 weeks), COVID-19 patients with 2-week convalescence phase; CP (6 months), COVID-19 patients with 6-month convalescence phase; TRA, T cell receptor α chain; TRB, T cell receptor β chain.

## Data Availability

The data that support the findings of this study are available from the corresponding author upon reasonable request.
